# Dietary Phytochemical Index and Fasting Plasma Glucose in Adults with Type 2 Diabetes: A Cross-Sectional Study

**DOI:** 10.3390/nu18193130

**Published:** 2026-09-23

**Authors:** Stavroula Maria Konstantopoulou, Alexandra Foscolou, Athanasia K. Papazafiropoulou, Anastasios Makris, Angeliki Dionysia Leftaki, George Panagiotopoulos, Aristea Gioxari, Dimitra Ioanna Lampropoulou, Paraskevi Detopoulou, Georgios I. Panoutsopoulos, Konstantina Argyri

**Affiliations:** 1 Department of Nutritional Science and Dietetics, School of Health Sciences, 24100 Kalamata, Greece; sm.konstantopoulou@go.uop.gr (S.M.K.); alexandra.foscolou@go.uop.gr (A.F.); adleftaki@yahoo.gr (A.D.L.); panagiotopoulos_g@yahoo.gr (G.P.); a.gioxari@go.uop.gr (A.G.); d.lambropoulou@go.uop.gr (D.I.L.); p.detopoulou@go.uop.gr (P.D.); gpanouts@go.uop.gr (G.I.P.); 2 First Department of Internal Medicine and Diabetes Center, Tzaneio General Hospital of Piraeus, 18536 Piraeus, Greece; pathan@ath.forthnet.gr; 3 Diabetes Clinic, General Hospital of Kalamata, 24100 Kalamata, Greece; tasomak@gmail.com

**Keywords:** type 2 diabetes mellitus, Dietary Phytochemical Index, dietary phytochemicals, fasting plasma glucose, glycemic control, glucose regulation, Mediterranean diet, dietary patterns, polyphenols

## Abstract

**Background/Objectives**: Type 2 diabetes mellitus (T2D) is a major public health challenge. Phytochemicals have gained attention for their potential role in glucose regulation. This study aimed to investigate the association between dietary phytochemical intake, assessed using the Dietary Phytochemical Index (DPI), and fasting plasma glucose in adults with established T2D. **Methods**: A cross-sectional study was conducted among 340 adults with T2D attending the Diabetes Clinic of the General Hospital of Kalamata, Greece. Dietary intake was assessed using a validated food frequency questionnaire and DPI was calculated as the percentage of total daily energy derived from fruits, vegetables, legumes, whole grains, nuts/seeds and olives/olive oil. Multivariable linear regression was used to examine the association between DPI and fasting plasma glucose, adjusting for demographic, lifestyle, anthropometric and clinical characteristics. The fully adjusted analysis included 199 participants with complete data. Additional models adjusted for Mediterranean diet adherence, total energy intake and socioeconomic factors. **Results**: Each 1-percentage-point increase in DPI was associated with 0.65 mg/dL lower fasting plasma glucose (b = −0.652, 95% CI: −1.177 to −0.127; and *p* = 0.015). The association remained after additional adjustment for Mediterranean diet adherence, total energy intake and socioeconomic factors and was supported by multiple-imputation analysis. Food-group component analyses were exploratory; no component remained statistically significant after false discovery rate correction. **Conclusions**: Higher DPI was associated with lower fasting plasma glucose in adults with T2D after adjustment for measured covariates. Prospective studies and randomized trials are needed to confirm this association and determine its clinical relevance.

## 1. Introduction

Diabetes mellitus is a group of metabolic disorders of carbohydrate metabolism that result in hyperglycemia [1]. Data from the International Diabetes Federation (IDF) highlight the growing impact of diabetes across the world, with an estimated 589 million adults aged 20–79 years living with diabetes in 2024. This number is projected to reach 853 million by 2050 [2]. A similar trend is expected in the IDF Europe Region, where the number of adults with diabetes is projected to increase by approximately 10% over the same period [2]. Type 2 diabetes (T2D) accounts for approximately 90–95% of all diabetes cases and currently ranks as the eighth leading cause of global disease burden and is estimated to become the second leading cause by 2050 [2]. It is characterized by relative insulin deficiency in association with insulin resistance [1]. Chronic inflammation, oxidative stress, β-cell dedifferentiation and persistent hyperglycemia contribute to disease progression and increase the risk of diabetes-related complications, including cardiovascular disease, diabetic kidney disease, retinopathy and neuropathy [3,4,5]. Taken together, these findings identify T2D as one of the major and rapidly growing global health challenges of the 21st century and underline the need for comprehensive management strategies to delay or prevent complications and improve quality of life [2].

Although nutrition is widely recognized as a cornerstone for T2D management, no single dietary pattern can be considered optimal for all individuals [6]. Current recommendations for people with T2D emphasize individualized and culturally appropriate eating patterns that support weight management and glycemic goals [7]. Healthful dietary patterns share several common characteristics, including non-starchy vegetables, whole fruits, legumes, whole grains, nuts and seeds and low-fat dairy products, while minimizing red meat, refined grains, added sugars and processed and ultra-processed foods [8,9]. Several dietary patterns are considered acceptable for diabetes management. Among these, Mediterranean, DASH, vegetarian and vegan dietary patterns have received particular attention [10,11] with their beneficial effects attributed to the synergistic action of dietary fiber, micronutrients, unsaturated fatty acids and bioactive compounds [12,13,14].

Phytochemicals, including polyphenols, alkaloids, terpenoids, phenolic acids, saponins and steroids, are bioactive compounds commonly found in plant-based foods that have attracted considerable interest for their potential role in the prevention and management of T2D, particularly through antioxidant, glucose-regulatory and anti-inflammatory mechanisms [15]. Oxidative stress is a key contributor to β-cell dysfunction, insulin resistance and diabetes-related complications and many phytochemicals act as antioxidants by scavenging reactive oxygen species, upregulating endogenous antioxidant defense enzymes and protecting pancreatic β-cells from oxidative damage. Concurrently, specific phytochemicals modulate carbohydrate-metabolizing enzymes (α-amylase, α-glucosidase), inhibit dipeptidyl peptidase-IV activity, enhance insulin secretion and improve peripheral glucose uptake. Furthermore, phytochemicals may reduce systemic low-grade inflammation that contributes to the development and progression of T2D and its microvascular and macrovascular complications by modulating inflammatory pathways, suppressing pro-inflammatory cytokine production (TNF-α, IL-1β and IL-6) and promoting anti-inflammatory immune responses [16,17,18]. Additionally, recent research suggests that phytochemicals may exert part of their antidiabetic effects through modulation of gut microbiota composition and function, thereby contributing to improved insulin sensitivity, glucose homeostasis and metabolic health [19].

Evidence from in vitro, animal and clinical studies suggests potential beneficial effects of isolated phytochemicals, phytochemical-rich foods and their extracts on glucose regulation and diabetes management [20,21,22]. However, foods are consumed as part of an overall dietary pattern rather than in isolation; therefore, it remains important to examine whether these associations are also observed in the context of habitual dietary intake. Furthermore, phytochemicals from different foods can interact additively or even synergistically, suggesting that the total dietary phytochemical content may influence glycemic control differently from the consumption of individual phytochemicals or single phytochemical-rich foods.

To address overall dietary exposure to phytochemical-rich foods, the Dietary Phytochemical Index (DPI) was proposed as the percentage of total daily energy intake derived from prespecified phytochemical-rich food sources [23]. In the present study, DPI included fruits, vegetables, legumes, whole grains, nuts/seeds and olives/olive oil. The DPI has attracted increasing scientific interest and has been associated with several cardiometabolic outcomes, including metabolic syndrome, hypertension and obesity [24,25,26,27,28,29], primarily in generally healthy populations or individuals at high metabolic risk. However, evidence regarding glycemic control in adults with established T2D remains scarce. Only a few observational studies have examined this association, with Li et al. [30] and Bahrampour et al. [31] reporting inverse associations between DPI and fasting plasma glucose, whereas Ziaee et al. reported no significant associations with fasting plasma glucose or glycated hemoglobin (HbA1c) [32]. These studies were conducted outside Europe. A Mediterranean setting is particularly relevant because several DPI components—including olive oil, fruits, vegetables, legumes, nuts/seeds and whole grains—are key components of the Mediterranean diet and customary foods within the region’s dietary tradition. Their relative contribution to DPI may therefore differ from that observed in other dietary settings, potentially influencing the composition and interpretation of the index across populations. Accounting for Mediterranean diet adherence may further clarify whether the association between DPI and glycemic status extends beyond overall adherence to the Mediterranean dietary pattern.

Glycemic indicators, including fasting plasma glucose, postprandial glucose and HbA1c, are central to the diagnosis and management of T2D, as they provide complementary information on glucose regulation and metabolic control [33]. Among these indicators, fasting plasma glucose reflects basal glucose regulation and is influenced by key mechanisms involved in T2D, including hepatic glucose production, insulin sensitivity and β-cell function, as well as by pharmacological treatment [34,35]. Its widespread availability, low cost and routine use in clinical practice make fasting plasma glucose a practical marker for examining potential associations between dietary factors and glycemic status in adults with T2D [34].

Therefore, the primary aim of this cross-sectional study was to investigate the association between DPI and fasting plasma glucose in adults with T2D from Messinia, Greece. Secondary objectives were to examine the association between DPI and HbA1c, as an indicator of longer-term glycemic exposure and the associations of individual phytochemical-rich food groups with fasting plasma glucose. Additional analyses examined the association between DPI and fasting plasma glucose after further adjustment for Mediterranean diet adherence and total energy intake.

## 2. Materials and Methods

### 2.1. Study Design and Participants

The present cross-sectional study is part of an ongoing observational study conducted in the region of Messinia, Greece, aiming to investigate the association of sociodemographic, lifestyle and nutritional characteristics with glycemic control in adults with T2D. Participants included in the present analysis were consecutively recruited from the Diabetes Clinic of the General Hospital of Kalamata between 27 May 2024 and 29 May 2026, during their scheduled follow-up visits. During these visits, the treating diabetologist assessed patients for eligibility according to the predefined inclusion and exclusion criteria. Eligible patients were then referred to the study team, informed about the purpose and procedures of the study and invited to participate. Those who agreed to participate provided written informed consent before enrollment. A total of 340 participants provided written informed consent, completed the study assessment and were included in the present analysis.

Eligible participants were adults (≥18 years) with an established diagnosis of T2D for at least 6 months, documented in the patients’ medical records and confirmed by the treating diabetologist, permanent residents of the Messinia region, attending the Diabetes Clinic of the General Hospital of Kalamata and able to provide written informed consent. Exclusion criteria included type 1 diabetes mellitus, gestational diabetes, pregnancy or lactation, inability to provide informed consent and conditions potentially affecting the reliability of glycemic measurements (e.g., hemoglobinopathies or anemia). Fasting plasma glucose was the primary glycemic outcome of the present analysis, while HbA1c was included as an additional glycemic marker.

### 2.2. Bioethics

The study protocol was approved by the Research Ethics Committee of the School of Health Sciences, University of Peloponnese (approval no. 4078/28.02.2024) and by the Scientific Council of the General Hospital of Kalamata (decision no. 20/24.05.2024). The study was conducted in accordance with the principles of the Declaration of Helsinki [36]. All participants were informed about the aims and procedures of the study and provided written informed consent prior to their enrollment. All data were anonymized before analysis, and no personal information was retained.

### 2.3. Measurements

Data were collected during clinic visits by trained personnel, including senior undergraduate students from the Department of Nutrition and Dietetics, registered dietitians and nurses. Sociodemographic, lifestyle, dietary and selected clinical information was obtained using a standardized, structured quantitative questionnaire, whereas anthropometric variables were directly measured using calibrated equipment and standardized measurement protocols.

#### 2.3.1. Sociodemographic Characteristics

The sociodemographic characteristics used in this study included age (years), sex (male/female), education (years of education) and financial status, as annual household income [Low (<€10,000), Moderate (€10,001–€20,000), Adequate (€20,001–€40,000) and High (>€40,000)].

#### 2.3.2. Lifestyle Characteristics

Lifestyle information included smoking and physical activity status. More specifically, participants who reported smoking at least one cigarette per day, occasional smoking or current vaping were classified as current smokers. Former smokers and never smokers were classified as non-smokers. Physical activity was evaluated in metabolic equivalent (MET) minutes per week, using the shortened, translated and validated-in-Greek version of the self-reported International Physical Activity Questionnaire (IPAQ) [37].

#### 2.3.3. Anthropometric and Clinical Characteristics

Body weight and height were measured using standard procedures. More specifically, body weight was measured to the nearest 0.1 kg and height to the nearest 0.1 cm. Body mass index (BMI), as body weight (kg)/height^2^ (m^2^), was then calculated. Waist and hip circumferences were assessed according to standardized anthropometric protocols, with participants wearing light clothing and no shoes. Waist circumference was measured to the nearest 0.1 cm using a non-stretchable measuring tape positioned midway between the lowest rib and the iliac crest. Hip circumference was measured to the nearest 0.1 cm in the horizontal plane at the level of the greater trochanters, corresponding to the widest part of the buttocks. The waist-to-hip ratio (WHR) was calculated by dividing waist circumference by hip circumference and was used as an indicator of central adiposity [38]. WHR values ≥ 0.90 in men and ≥0.85 in women were considered substantially increased [39].

Data regarding T2D duration (years since diagnosis) and current antidiabetic treatment, categorized as oral antidiabetic treatment, insulin therapy, or combination therapy, were obtained. Participants reported the presence of selected chronic diseases or health-related problems, including hypertension, dyslipidemia, renal dysfunction, respiratory disease, cardiovascular disease, depression or anxiety disorder, cancer, arthritis, osteoporosis, tremor or Parkinson’s disease, multiple sclerosis, gastrointestinal problems, gastroesophageal reflux disease, frequent urinary tract infections, mobility problems, thyroid disorders, sleep problems, swallowing difficulties, chewing difficulties and/or vision problems. Diabetes was not included as a separate health condition, since all participants had been diagnosed with diabetes. Fasting plasma glucose (mg/dL) and glycated hemoglobin (HbA1c) were obtained from the most recent available laboratory test results performed within one month before enrollment. Laboratory measurements were not performed in a single laboratory and detailed information on the analytical methods used was not consistently available. Laboratory fasting glucose measurements were based on venous blood sampling. When recent laboratory fasting glucose results were unavailable, fasting glucose was measured during the clinic visit at the Diabetes Clinic of the General Hospital of Kalamata from capillary whole blood using an Exactive Vital+ blood glucose meter (Model 1004+; MicroTech Medical Co., Ltd., Hangzhou, China). This procedure was applied to 19 (8.8%) participants. According to the manufacturer’s technical documentation, the device is calibrated against a plasma reference and reports plasma-equivalent glucose concentrations [40]. If participants had not fasted for at least 8 h before the clinic visit, fasting plasma glucose was not measured and was therefore recorded as missing.

#### 2.3.4. Dietary Characteristics

Dietary habits, total daily energy intake and macronutrient intake (total daily carbohydrate, protein and fat intake) were assessed using a validated semi-quantitative food frequency questionnaire (FFQ) [41], with participants instructed to report their usual food consumption over the preceding month. Participants were asked to report the consumption (never or rarely, 1–3 times/month, 1–2 times/week, 3–6 times/week, once/day, and more than 2/day) of a variety of food groups and beverages, including meat and meat products, poultry, fish, milk or other dairy products, fruits, vegetables, legumes and alcohol. The MedDietScore [42] was employed to assess the adherence to the Mediterranean diet with a theoretical range of 0 to 55. Higher values imply higher adherence [42].

Based on FFQ data, the Dietary Phytochemical Index (DPI) [43] was calculated as 100 × (daily energy derived from the phytochemical-rich food groups included in the present FFQ/total daily energy intake) [23,43]. In the present study, the available DPI components were fruits, vegetables, legumes, whole grains, nuts/seeds and olives/olive oil. The numerator was the sum of energy derived from these six components. The fruit component included whole fruits, fresh fruit juice and dried fruit. Nuts and seeds were combined because they were assessed within the same FFQ item, while olives and olive oil were combined to represent energy derived from olives and olive-derived products. Soy products were not captured by the FFQ and therefore did not contribute to the DPI calculation. Thus, the DPI used in the present study was modified to reflect the phytochemical-rich food groups available from the FFQ. Higher DPI values indicate a greater contribution of phytochemical-rich foods to total energy intake.

### 2.4. Sample Size

No formal a priori sample-size calculation was performed, as the sample size was determined by the number of eligible participants enrolled during the recruitment period. The total study sample comprised 340 participants; however, owing to missing data, the primary fully adjusted complete-case analysis included 199 participants. An a posteriori sensitivity calculation indicated that this analytical sample provided 80% power, at a two-sided α of 0.05, to detect an absolute correlation of approximately |r| = 0.20. This calculation is provided to describe the detectable effect size and should not be interpreted as evidence that the study was adequately powered for all secondary analyses.

### 2.5. Statistical Analysis

Continuous variables are presented as mean ± standard deviation (SD), whereas categorical variables are presented as frequencies and percentages. The normality of continuous variables was assessed using the Kolmogorov–Smirnov test, along with visual inspection of histograms and Q–Q plots. Participants were stratified into four DPI groups (i.e., Q1, Q2, Q3, and Q4) according to DPI quartiles of the total sample. Continuous variables were compared using analyses of variance (ANOVA), while chi-square test was used to investigate associations between categorical variables. Post hoc pairwise comparisons between groups were performed using Tukey’s HSD test. To assess potential selection bias associated with the complete-case approach, the characteristics of participants included in the primary analytic sample were compared with those of participants not included because of incomplete data. Continuous variables were compared using independent-sample *t*-test, whereas categorical variables were compared using Pearson’s chi-square tests. The correlation between continuous DPI and fasting plasma glucose was evaluated using Pearson’s correlation coefficient. Regression analyses were based on complete cases for the variables included in each model. Accordingly, the analytical sample varied across models. No imputation of missing outcome or covariate data was performed for the primary analyses. Participants included in the fully adjusted complete-case model were compared with those not included with respect to demographic, lifestyle, anthropometric, clinical, glycemic and dietary characteristics. The primary association between continuous DPI and fasting plasma glucose was examined using linear regression. Model 1 was crude; Model 2 adjusted for age and sex; Model 3 additionally adjusted for BMI, physical activity and smoking/vaping status; and Model 4 additionally adjusted for diabetes duration, antidiabetic treatment category and number of health conditions. Antidiabetic treatment was entered as two indicator variables (insulin and combination therapy), with oral treatment as the reference. Covariates were selected a priori because of their plausible relations with diet and glycemia. In addition, sensitivity Model 5a added MedDietScore, Model 5b added total energy intake and Model 5c added both variables simultaneously. A further socioeconomic sensitivity model (Model 6) was additionally adjusted for years of education and financial status. DPI quartiles were also examined as a categorical exposure in linear regression models, with Q1 as the reference category. Linear trend across DPI quartiles was assessed by assigning each participant the median DPI value of their quartile and entering this value as a continuous term in the regression model. HbA1c was examined as an additional glycemic marker using the same covariate sequence. Individual DPI food-group analyses were treated as exploratory and Benjamini–Hochberg false discovery rate (BH-FDR) correction was applied across the six component tests. As a sensitivity analysis addressing potential dietary energy misreporting, the primary regression model was repeated after excluding participants with extreme reported energy intakes (<500 or >3500 kcal/day for women and <800 or >4200 kcal/day for men), using predefined sex-specific energy-intake cut-offs [44]. Regression assumptions were evaluated using model-based diagnostic procedures. Linearity and homoscedasticity were assessed by visual inspection of standardized residuals versus standardized predicted values. For the primary DPI exposure, potential departure from linearity was additionally examined by including a mean-centered quadratic DPI term in the fully adjusted model. Residual normality was evaluated using histograms and Q–Q plots of standardized residuals. Potentially influential observations were assessed using standardized residuals, leverage values, and Cook’s distance. Multicollinearity was evaluated using tolerance and variance inflation factor (VIF) statistics, with tolerance values < 0.20 and VIF values ≥ 5 considered indicative of potentially problematic multicollinearity [45]. Bias-corrected and accelerated 95% bootstrap confidence intervals based on 5000 resamples were calculated to assess the robustness of the regression estimates. R^2^ and adjusted R^2^ were recorded for all models. To assess the robustness of the complete-case findings to missing data, a sensitivity analysis was performed using multiple imputation by chained equations under the missing-at-random assumption. Fifty imputed datasets were generated, using 20 iterations per dataset. The imputation model included fasting plasma glucose, HbA1c, DPI and all demographic, lifestyle, anthropometric, clinical, dietary and socioeconomic variables included in the analytical models. Continuous variables were imputed using predictive mean matching, binary variables using logistic regression and nominal categorical variables using polytomous logistic regression. The regression models were repeated in each imputed dataset, and coefficients and standard errors were pooled using Rubin’s rules. All statistical analyses were performed using the R software, v.4.6.1 (R Foundation for Statistical Computing, Vienna, Austria) and SPSS software, version 31.0 (IBM Corp., Armonk, NY, USA). A two-sided *p* value < 0.05 was considered statistically significant.

## 3. Results

A total of 340 participants completed the study assessment. Fasting plasma glucose was available for 216 participants, whereas HbA1c was available for 330 participants. The primary fully adjusted fasting-glucose model included 199 complete cases; thus, 17 additional participants with available fasting glucose were excluded because of missing data in one or more covariates. Table 1 presents the socio-demographic, lifestyle, anthropometric, clinical and dietary characteristics of the 340 adults with T2D according to DPI quartiles [i.e., Q1 (DPI < 41.3%, n = 85), Q2 (DPI between 41.3 and 50%, n = 85), Q3 (DPI between 50 and 58.4%, n = 85) and Q4 (DPI > 58.4%, n = 85)]. The mean age of the total sample was 65.9 ± 11.4 years and 49.7% were female. Fasting plasma glucose progressively decreased across increasing DPI quartiles, from 141 ± 53 mg/dL in Q1 to 120 ± 27 mg/dL in Q4 (*p* = 0.048), whereas HbA1c was similar across quartiles (*p* = 0.480). MedDietScore ranged from 29.9 ± 5.2 in Q1 to 31.4 ± 3.8 in Q4, without a statistically significant overall difference across quartiles (*p* = 0.054). Age, sex, education, smoking/vaping status, physical activity, diabetes duration, antidiabetic treatment and number of health conditions were similar across DPI quartiles (all *p*’s > 0.05). BMI varied across DPI quartiles (Q1: 31.0 ± 6.4, Q2: 29.4 ± 5.5, Q3: 31.9 ± 7.5 and Q4: 29.7 ± 5.7 kg/m^2^; *p* = 0.029), while WHR was similar across quartiles (*p* = 0.550). Total daily energy, carbohydrate, protein and fat intakes generally decreased across increasing DPI quartiles (all *p*’s < 0.001), with the corresponding quartile-specific values reported in Table 1. The characteristics of participants included in the primary complete-case analysis (n = 199) and those not included because of incomplete data (n = 141) are presented in Supplementary Table S1. No statistically significant between-group differences were detected in the examined sociodemographic, lifestyle, anthropometric, clinical or dietary characteristics.

To distinguish energy amount from dietary composition, macronutrients expressed as percentage of energy and absolute energy derived from each DPI component are additionally reported in Supplementary Table S2. With increasing DPI quartiles, the percentages of energy derived from carbohydrate and protein decreased, while the percentage derived from fat increased (all *p* < 0.001). The absolute energy contribution of the combined DPI food groups increased progressively from 1084 ± 400 kcal/day in Q1 to 1393 ± 281 kcal/day in Q4 (*p* < 0.001). Among individual DPI components, absolute energy derived from whole grains (*p* = 0.016) and olives/olive oil (*p* < 0.001) differed significantly across quartiles, whereas the corresponding values for fruits, vegetables, legumes and nuts/seeds were similar across quartiles (Supplementary Table S2).

DPI (continuous variable) was inversely correlated with fasting plasma glucose (r = −0.182, *p* = 0.009). The primary association between DPI and fasting plasma glucose was subsequently examined using multivariable linear regression models.

As depicted in Table 2, higher DPI was associated with lower fasting plasma glucose across all models (Model 1–4). More specifically, in the fully adjusted model (Model 4), each one-percentage-point higher DPI was associated with a 0.652 mg/dL lower fasting plasma glucose concentration (b = −0.652, 95% CI: −1.177 to −0.127, and *p* = 0.015), after adjustment for age, sex, BMI, physical activity, smoking/vaping status, diabetes duration, antidiabetic treatment and health conditions. Accordingly, a 10-percentage-point higher DPI was associated with approximately 6.5 mg/dL lower fasting plasma glucose (Model 4). The estimate was also supported by BCa bootstrap analysis based on 5000 resamples (95% CI: −1.318 to −0.163). The association remained after adding MedDietScore (Model 5a: b = −0.610, 95% CI: −1.152 to −0.069; *p* = 0.027), total energy intake (Model 5b: b = −0.763, 95% CI: −1.370 to −0.157; *p* = 0.014), and both MedDietScore and total energy intake simultaneously (Model 5c: b = −0.713, 95% CI: −1.358 to −0.068; *p* = 0.030). Additional adjustments for education and financial status revealed a similar estimate (Model 6: b = −0.722, 95% CI: −1.249 to −0.196; *p* = 0.007). However, no statistically significant association between DPI and HbA1c was observed in the fully adjusted model (Model 4: b = −0.003, 95% CI: −0.014 to 0.008; *p* = 0.598), or in the additional sensitivity models including Mediterranean diet adherence, total energy intake, both variables simultaneously or socioeconomic indicators (all *p*’s > 0.05). As a sensitivity analysis addressing potential energy misreporting, the primary fully adjusted model was repeated after excluding participants with extreme reported energy intakes (<500 or >3500 kcal/day for women and <800 or >4200 kcal/day for men). Following exclusion of 29 participants with implausible energy intake, 311 participants remained eligible for the sensitivity analysis, of whom 172 had complete data for all variables included in the model. The inverse association between DPI and fasting plasma glucose remained statistically significant (b = −0.827, 95% CI: −1.422 to −0.233, and *p* = 0.007) (Supplementary Table S3).

Regression diagnostics showed no evidence of non-linearity for the main DPI association (quadratic DPI term, *p* = 0.688). Some deviation from normality was observed in the residual histogram and Q–Q plot, mainly in the upper tail, and the residual-versus-predicted plot suggested some heteroscedasticity. Five observations had standardized residuals greater than three. However, no observation had a Cook’s distance above one (maximum = 0.260), while the maximum leverage value was 0.315.

The complete-case findings were supported by the multiple-imputation sensitivity analysis (Supplementary Table S4). In the fully adjusted model including all 340 participants across 50 imputed datasets, each one-percentage-point higher DPI was associated with 0.518 mg/dL lower fasting plasma glucose (pooled b = −0.518, 95% CI: −0.958 to −0.079; *p* = 0.021), compared with 0.652 mg/dL in the complete-case analysis (b = −0.652, SE = 0.266, 95% CI: −1.177 to −0.127; *p* = 0.015). Thus, the magnitude of the association was modestly attenuated, while the direction of the association was unchanged and remained statistically significant.

When DPI was examined in quartiles (Q1–Q4) (Table 3), participants in the highest DPI quartile (Q4) exhibited significantly lower fasting plasma glucose concentrations compared with those in the lowest quartile (Q1) in the fully adjusted model. More specifically, participants in Q4 had 20.9 mg/dL lower fasting plasma glucose than those in Q1 (b = −20.9, 95% CI: −38.87 to −2.93, and *p* = 0.023). The corresponding estimates were −6.12 mg/dL for Q2 (95% CI: −24.90 to 12.65; *p* = 0.521) and −15.23 mg/dL for Q3 (95% CI: −33.34 to 2.87; *p* = 0.099). A test assigning the median DPI of each quartile as a continuous score supported a graded association (b = −0.771 mg/dL per one-percentage-point increase in the quartile median, 95% CI: −1.383 to −0.158, and *p*-trend = 0.014).

Similarly, these findings were supported by the multiple-imputation sensitivity analysis (Supplementary Table S5). Participants in Q4 had lower fasting plasma glucose than those in Q1 (pooled b = −15.9 mg/dL, 95% CI: −30.8 to −0.94; *p* = 0.037), and the linear trend across quartiles remained statistically significant (pooled b = −0.60, 95% CI: −1.11 to −0.09; *p*-trend = 0.021). The linear trend across quartiles also remained statistically significant after excluding participants with implausible reported energy intakes (pooled b = −0.59, 95% CI: −1.07 to −0.11; *p* = 0.017).

The six individual DPI food-group components were examined on an exploratory basis. In separate adjusted models (Table 4), fruit (b = −1.830, 95% CI: −3.801 to 0.141; *p* = 0.069) and whole-grain energy contribution (b = −1.875, 95% CI: −3.904 to 0.154; *p* = 0.070) showed inverse point estimates, although neither association was statistically significant. None of the six component associations remained statistically significant after Benjamini–Hochberg FDR correction (all q ≥ 0.203).

Furthermore, when all DPI food-group components were entered simultaneously into the same mutually adjusted multivariable linear regression analysis (Figure 1), none showed a statistically significant association with fasting plasma glucose after adjustments. Estimates were: fruits b = −1.878 (95% CI: −3.875 to 0.118; *p* = 0.065), vegetables b = 0.482 (95% CI: −2.293 to 3.258; *p* = 0.732), legumes b = 0.316 (95% CI: −2.287 to 2.919; *p* = 0.811), whole grains b = −1.881 (95% CI: −3.978 to 0.216; *p* = 0.078), nuts/seeds b = −0.352 (95% CI: −2.076 to 1.371; *p* = 0.687), and olives/olive oil b = −0.540 (95% CI: −1.226 to 0.145; *p* = 0.122). None of the component associations remained statistically significant after FDR correction (all q’s between 0.235 and 0.811).

## 4. Discussion

The present study investigated the association between habitual phytochemical intake, as assessed by the DPI and fasting plasma glucose among adults with established T2D in Messinia, a region of the Peloponnese, Greece.

The most important finding of this study was that higher DPI was associated with lower fasting plasma glucose in adults with T2D. In the fully adjusted model, a one-percentage-point difference in DPI corresponded to approximately 0.65 mg/dL lower fasting plasma glucose (b = −0.652, 95% CI: −1.177 to −0.127); thus, a 10-percentage-point higher DPI corresponded to approximately 6.5 mg/dL lower fasting plasma glucose. The magnitude of this association may be clinically meaningful; however, given the cross-sectional design of the study, it should not be interpreted as an expected treatment effect. This association persisted after adjustment for demographic, lifestyle and clinical characteristics and was further supported by the quartile analysis, in which participants in the highest DPI quartile had lower fasting plasma glucose concentrations than those in the lowest quartile. Previous studies investigating the association between DPI and glycemic parameters have yielded inconsistent findings, although methodological differences across studies limit direct comparisons. In the Swiss CoLaus study, Gamba et al. included 3879 adults from the general population (53.5% women; mean age 57.6 years) and reported a median DPI of 25.5 (IQR: 17.7–34.6); no significant association between DPI and fasting plasma glucose was observed after multivariable adjustment [27]. In contrast, studies conducted in adults with T2D have generally included smaller populations. Ziaee et al. studied 235 Iranian adults with T2D (aged 30–65 years; diabetes duration 1–10 years) using three 24 h dietary recalls and found no significant correlation between DPI and fasting glucose or HbA1c [32]. Bahrampour et al. examined 210 Iranian women with T2D in a case–control study of diabetic nephropathy, using a validated 147-item FFQ and categorizing DPI by the median; women with higher DPI had lower fasting glucose in the nephropathy group, although glycemic parameters were not the primary outcomes [31]. More recently, Li et al. studied 675 Chinese adults with T2D (mean age 60.1 years, 48.1% women; mean diabetes duration 5.8 years) using a validated FFQ. Mean DPI was 38.5 ± 8.9%, ranging from 27.5% (Q1) to 49.7% (Q4). Lower fasting glucose, HbA1c and HOMA-IR were observed across higher DPI quartiles [30]. The present study included 340 adults with established T2D (mean age 65.9 years, 50.3% men; mean diabetes duration 12.2 years). Fasting plasma glucose was available for 216 participants, of whom 199 were included in the primary fully adjusted complete-case analysis. Mean DPI was 50.0 ± 12.0%, with quartile cut-offs ranging from <41.3% (Q1) to >58.4% (Q4). Dietary intake was assessed using a validated FFQ and DPI was analyzed both continuously and across quartiles, with adjustment for age, sex, lifestyle, anthropometric and clinical factors, including diabetes duration and antidiabetic treatment. Differences in study populations, dietary assessment methodology, operational definition of DPI, diabetes duration, glycemic outcomes and covariate adjustment may partly explain the inconsistent findings across studies.

Several biologically plausible mechanisms proposed in previous studies may contribute to the interpretation of the observed inverse association between DPI and fasting plasma glucose. Phytochemicals, including polyphenols, carotenoids, tannins and saponins, are abundant in plant-based foods such as fruits, vegetables, legumes and whole grains which are major components of the DPI. Previous studies suggest that these compounds may influence glucose metabolism through multiple mechanisms, including: (i) enhancing beta-cell function, (ii) enhancing antioxidant defense and reducing inflammatory response, (iii) improving insulin sensitivity, (iv) inhibiting carbohydrate-digesting enzymes such as α-amylase and α-glucosidase, (v) reducing hepatic glucose production and increasing glucose uptake in adipocytes and (vi) improving gut health and promoting beneficial microbiota [20,21]. It should also be acknowledged that phytochemical-rich diets are naturally high in dietary fiber, which may contribute to the observed association. A recent meta-analysis of randomized controlled trials demonstrated that dietary fiber intake significantly reduced fasting blood glucose, HbA1c, fasting insulin and insulin resistance in adults with T2D [46]. Therefore, the metabolic benefits associated with a higher DPI are likely to reflect the combined effects of phytochemicals and other bioactive food components. Whole fruits, vegetables, legumes, nuts and olive oil provide complex food matrices in which polyphenols interact with dietary fiber, vitamins and other micronutrients, potentially enhancing their metabolic effects [47].

The second important finding of the present study was that the inverse association between DPI and fasting plasma glucose remained statistically significant after adjustment for adherence to the Mediterranean dietary pattern. The Mediterranean diet is one of the most extensively studied dietary patterns for the prevention and management of type 2 diabetes and its beneficial effects have been partly attributed to its high content of phytochemical-rich foods and their antioxidant and anti-inflammatory properties [48]. Consistent with these observations, total dietary antioxidant capacity has also been inversely associated with fasting glucose in a Greek population, although these associations were observed primarily among participants with lower adherence to the Mediterranean dietary pattern [49]. Interestingly, in our study, the persistence of the association between DPI and fasting plasma glucose after adjustment for MedDietScore suggests that this association may not be entirely accounted for by adherence to the Mediterranean diet. One possible explanation is that although both indices reflect diet quality, the DPI specifically captures the contribution of phytochemical-rich foods to the overall diet. The inverse association between DPI and fasting plasma glucose remained statistically significant after adjustment for total energy intake. Moreover, the association between DPI and fasting plasma glucose remained statistically significant when Mediterranean diet adherence and total energy intake were simultaneously controlled in a separate sensitivity model.

The third finding of the present study was that DPI was inversely associated with fasting plasma glucose but not with HbA1c. This finding is consistent with the results of the study of Bahrampour et al. [31], who also reported an inverse association between DPI and fasting plasma glucose, whereas no significant association was observed with HbA1c among adults with T2D. Previous research suggests that one possible explanation for the observed association is that phytochemical-rich diets may influence fasting glucose through pathways involving insulin sensitivity, oxidative stress, inflammation and hepatic glucose regulation. In contrast, HbA1c reflects average glycemic exposure over the preceding 2–3 months and integrates both fasting and postprandial glucose levels [50,51]. Therefore, the effects of dietary factors influencing specific aspects of glucose regulation may not be equally reflected by fasting plasma glucose and HbA1c.

An important consideration when interpreting these findings is that DPI provides an estimate of dietary phytochemical composition. Because DPI is based on the proportion of energy derived from phytochemical-rich foods, it may be more strongly influenced by energy-dense plant sources. The substantial decrease in total energy intake across increasing DPI quartiles should also be considered when interpreting the findings. In addition, the absolute energy contribution of DPI food groups increased across quartiles and the inverse association between DPI and fasting plasma glucose persisted after adjustment for total energy intake. Moreover, the index does not account for differences in phytochemical concentration, diversity or bioavailability. Despite these limitations, DPI has been increasingly used in observational studies investigating associations with chronic conditions, including stroke, hypertension, cancer, obesity and osteoporosis, supporting its utility as a practical dietary index [29].

There are several strengths of the present study that should be acknowledged. To our knowledge, this is the first study conducted in a European Mediterranean population with established T2D to assess the association between DPI and fasting plasma glucose. This addresses an important research gap, as previous studies have predominantly been conducted in Asian populations and evidence from Mediterranean populations remains limited. Furthermore, to our knowledge, this is the first study to examine the association between DPI and fasting plasma glucose after accounting for overall diet quality, as assessed by adherence to the Mediterranean diet. This study included a well-characterized sample of adults with established T2D and incorporated adjustments for potential confounding factors, including sociodemographic, lifestyle, anthropometric, clinical and dietary characteristics. Moreover, the study examined the contribution of individual phytochemical-rich food groups. The robustness of the findings was further evaluated through sensitivity analyses accounting for Mediterranean diet adherence, total energy intake, socioeconomic factors and implausible reported energy intakes as well as through bootstrap estimation and multiple imputation for missing data.

However, several limitations should also be considered. Due to the cross-sectional design of the study, causality cannot be established and it cannot be determined whether higher DPI preceded lower fasting plasma glucose. Reverse causation cannot be excluded, as participants may have modified their dietary habits following medical or nutritional counseling in response to poor glycemic status, while those with better-managed diabetes may also exhibit generally healthier dietary and lifestyle behaviors. A systematic screening log was not maintained; therefore, complete pre-enrollment screening and recruitment data were unavailable. Additionally, although 340 participants were enrolled, fasting plasma glucose was available for 216 and the primary fully adjusted analysis included 199 complete cases. Therefore, the analytical sample may not fully represent all enrolled participants. Dietary intake was assessed using a self-reported food frequency questionnaire, which is subject to recall bias and measurement error. Differential energy intake under-reporting cannot be excluded and may have influenced DPI, given that total energy intake is included in the denominator of the index. In addition, soy intake was not captured by the FFQ and therefore was not included in the DPI calculation. This may have resulted in underestimation of DPI and may limit direct comparisons with studies in which soy was included in the index. Furthermore, glycemic parameters were obtained either from recent laboratory results or during the clinic visit and laboratory measurements were not performed in a single laboratory. This may have introduced some measurement variability. In addition, fasting plasma glucose was based on a single measurement and may therefore not fully reflect usual glycemic status. Detailed information on specific antidiabetic medication classes, dosage, treatment intensity, adherence and recent treatment changes was not systematically available; therefore, residual confounding related to pharmacological treatment cannot be excluded. Residual confounding from other unmeasured dietary, behavioral or clinical factors cannot be completely excluded. Although no statistically significant differences were observed between participants included and not included in the complete-case analysis, potential bias due to missing data cannot be excluded. Multiple statistical comparisons were also performed, which may have increased the probability of chance findings, although correction for multiple testing was applied to the exploratory food-group analyses. Finally, the generalizability of the findings may be limited because the study population consisted of adults with T2D from a single region of Greece.

Overall, the findings of the present study provide a basis for future research investigating the role of dietary phytochemical intake in glycemic control among individuals with T2D. Prospective cohort studies and randomized controlled trials are needed to determine whether increasing the consumption of phytochemical-rich foods can lead to sustained improvements in fasting plasma glucose and other markers of glycemic control. Future studies should also investigate potential sex differences in the association between DPI and glycemic control, as well as the contribution of specific phytochemical-rich food groups and the underlying biological mechanisms.

## 5. Conclusions

In conclusion, a higher DPI was associated with lower fasting plasma glucose among adults with established T2D. This association persisted after accounting for Mediterranean diet adherence and total energy intake. In contrast, no significant association was observed between DPI and HbA1c. The observed magnitude of the association suggests that phytochemical-rich dietary patterns may be relevant to glycemic status; however, given the cross-sectional design of the study, the findings are not sufficient to support DPI-based dietary strategies in the clinical management of T2D. Prospective studies and randomized controlled trials are needed to confirm these findings and further investigate the potential role of phytochemical-rich dietary patterns in glycemic control among adults with T2D.

## Figures and Tables

**Figure 1:**
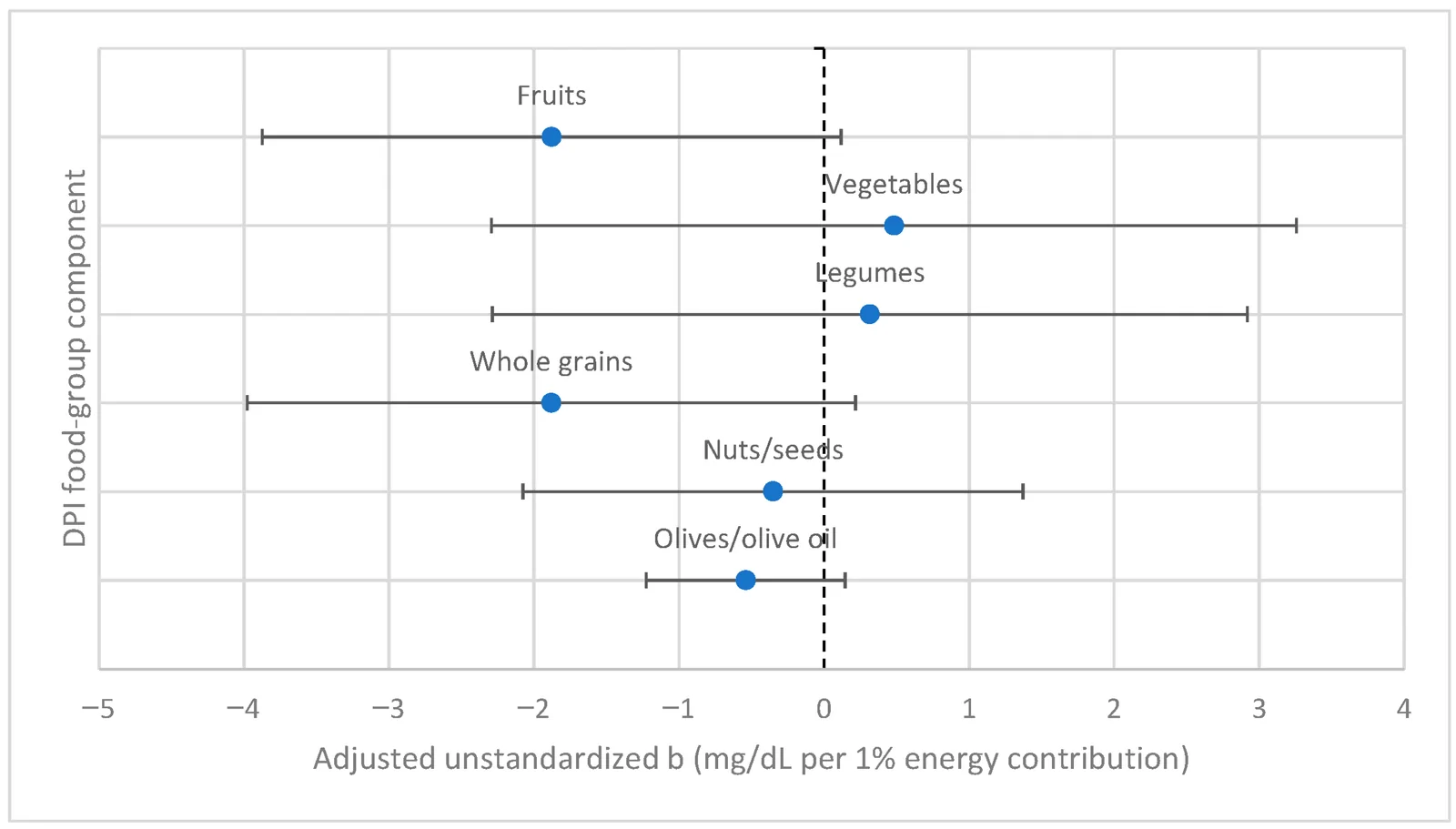
Exploratory mutually adjusted associations between DPI food-group components and fasting plasma glucose. Analytical sample = 199. All food-group components were entered simultaneously in the same multivariable model, adjusted for age, sex, BMI, physical activity, smoking/vaping status, diabetes duration, antidiabetic treatment (dummy coded with oral treatment as reference) and health conditions. No evidence of problematic multicollinearity was observed among the six DPI food-group components (VIF range: 1.060–1.205 and tolerance range: 0.830–0.943). Points represent adjusted unstandardized b-coefficients and horizontal bars represent 95% confidence intervals.

**Table 1:** Basic socio-demographic, lifestyle, anthropometric, clinical and dietary characteristics of the total sample (N = 340 individuals with type 2 diabetes mellitus) by Dietary Phytochemical Index quartiles.

Characteristics	Total Sample (N = 340)	Q1 (DPI < 41.3%) (n = 85)	Q2 (DPI 41.3–50%) (n = 85)	Q3 (DPI 50–58.4%) (n = 85)	Q4 (DPI > 58.4%) (n = 85)	*p*
Socio-demographic characteristics						
Age (years)	65.9 ± 11.4	64.0 ± 11.9	66.0 ± 11.3	66.8 ± 12.0	66.8 ± 10.4	0.348
Males, n (%)	171 (50.3)	44 (51.8)	50 (58.8)	43 (50.6)	34 (40.0)	0.104
Education (years)	10.7 ± 4.3	10.9 ± 4.6	10.4 ± 4.2	10.9 ± 4.1	10.5 ± 4.4	0.816
Financial status % (Low/Moderate/Adequate/High)	35.1/48.7/15.3/0.9	39.3/48.8/10.7/1.2	28.2/54.1/17.6/0.0	40.0/44.7/14.1/1.2	32.9/47.1/18.8/1.2	0.708
Lifestyle characteristics						
Smoking, yes n (%)	99 (29.1)	24 (28.2)	31 (36.5)	22 (25.9)	22 (25.9)	0.336
IPAQ (MET-min/week)	743 ± 1008	861 ± 1192	706 ± 851	644 ± 916	760 ± 1042	0.554
Anthropometric and clinical characteristics						
BMI (kg/m^2^)	30.5 ± 6.4	31.0 ± 6.4	29.4 ± 5.5	31.9 ± 7.5	29.7 ± 5.7	**0.029**
Waist-to-hip ratio	0.97 ± 0.11	0.99 ± 0.10	0.96 ± 0.10	0.97 ± 0.10	0.95 ± 0.13	0.550
For males	1.02 ± 0.08	1.03 ± 0.08	0.98 ± 0.09	1.01 ± 0.08	1.06 ± 0.08	0.259
For females	0.91 ± 0.10	0.93 ± 0.11	0.94 ± 0.10	0.91 ± 0.08	0.90 ± 0.11	0.819
Diabetes duration (years)	12.2 ± 9.3	11.0 ± 8.0	13.7 ± 9.4	11.6 ± 9.1	12.5 ± 10.3	0.271
Antidiabetic treatment % (oral antidiabetic treatment/insulin/combination therapy)	65.8/7.4/26.8	61.2/9.4/29.4	64.7/9.4/25.9	68.2/5.9/25.9	69.0/4.8/26.2	0.837
Fasting plasma glucose (mg/dL)	133 ± 45 (n = 216)	141 ± 53 (n = 65)	139 ± 54 (n = 49)	126.8 ± 34 (n = 53)	120 ± 27 (n = 49)	**0.048**
HbA1c (%)	6.8 ± 1.3 (n = 330)	6.9 ± 1.6 (n = 83)	6.9 ± 1.4 (n = 81)	6.6 ± 0.8 (n = 85)	6.9 ± 1.5 (n = 81)	0.480
Health conditions (number)	2.6 ± 1.8	2.4 ± 1.8	2.7 ± 1.7	2.8 ± 1.9	2.4 ± 1.8	0.496
Dietary characteristics						
MedDietScore (0–55)	30.7 ± 4.4	29.9 ± 5.2	30.2 ± 4.2	31.3 ± 4.4	31.4 ± 3.8	0.054
DPI (%)	50 ± 12	34.6 ± 5.7 ^b,c,d^	46.1 ± 2.5 ^a,c,d^	54.0 ± 2.5 ^a,b,d^	65 ± 5.5 ^a,b,c^	**<0.001**
Carbohydrate intake (g/d)	196 ± 84	252 ± 104 ^b,c,d^	212 ± 72 ^a,c,d^	173 ± 59 ^a,b,d^	148 ± 48 ^a,b,c^	**<0.001**
Protein intake (g/d)	119 ± 52	151 ± 65 ^b,c,d^	129 ± 52 ^a,c,d^	105 ± 32 ^a,b^	89 ± 25 ^a,b^	**<0.001**
Fat intake (g/d)	157 ± 45	172 ± 60 ^c,d^	163 ± 42 ^d^	152 ± 37 ^a^	140 ± 27 ^a,b^	**<0.001**
Total energy intake (kcal/d)	2620 ± 814	3120 ± 1047 ^b,c,d^	2780 ± 721 ^a,c,d^	2438 ± 569 ^a,b,d^	2142 ± 438 ^a,b,c^	**<0.001**

Data are presented as mean ± standard deviation for continuous variables and as frequencies and percentages for categorical variables. Differences across DPI quartiles were assessed using one-way analysis of variance (ANOVA) for continuous variables and the chi-square test for categorical variables. Statistical significance was set at *p*-value < 0.05, marked with bold. DPI: Dietary Phytochemical Index; IPAQ: International Physical Activity Questionnaire; BMI: body mass index; HbA1c: glycated hemoglobin. ^a^ Statistically significant comparison with Q1; ^b^ statistically significant comparison with Q2; ^c^ statistically significant comparison with Q3; ^d^ statistically significant comparison with Q4. Post hoc pairwise comparisons for continuous variables were performed using Tukey’s HSD test.

**Table 2:** Multiple linear regression models assessing the association of Dietary Phytochemical Index (DPI), modeled as a continuous variable, with fasting plasma glucose (primary outcome) and HbA1c (secondary outcome) among adults with type 2 diabetes.

Outcome	Sample (n)	Models	b ± SE per 1% Increase in DPI	95% CI	R^2^	Adjusted R^2^	*p*-Value
Fasting glucose	216	Model 1 (crude): DPI	−0.668 ± 0.253	−1.167; −0.170	0.033	0.028	**0.009**
	215	Model 2: Model 1 + age, sex	−0.674 ± 0.256	−1.179; −0.168	0.037	0.023	**0.009**
	209	Model 3: Model 2 + BMI, physical activity, smoking/vaping status	−0.700 ± 0.260	−1.213; −0.188	0.048	0.018	**0.008**
	199	Model 4: Model 3 + diabetes duration, antidiabetic treatment, health conditions	−0.652 ± 0.266	−1.177; −0.127	0.093	0.042	**0.015**
	199	Model 5a (sensitivity): Model 4 + MedDietScore	−0.610 ± 0.274	−1.152; −0.069	0.095	0.039	**0.027**
	199	Model 5b (sensitivity): Model 4 + total energy intake	−0.763 ± 0.308	−1.370; −0.157	0.096	0.04	**0.014**
	199	Model 5c (sensitivity): Model 4 + MedDietScore + total energy intake	−0.713 ± 0.327	−1.358; −0.068	0.097	0.036	**0.03**
	199	Model 6 (sensitivity): Model 4 + education and financial status	−0.722 ± 0.267	−1.249; −0.196	0.084	0.014	**0.007**
HbA1c (%)	330	Model 1 (crude): DPI	−0.004 ± 0.006	−0.016; 0.009	0.001	−0.002	0.577
	328	Model 2: Model 1 + age, sex	−0.006 ± 0.006	−0.017; 0.005	0.019	0.010	0.287
	320	Model 3: Model 2 + BMI, physical activity, smoking/vaping status	−0.006 ± 0.006	−0.017; 0.006	0.020	0.001	0.329
	310	Model 4: Model 3 + diabetes duration, antidiabetic treatment, health conditions	−0.003 ± 0.006	−0.014; 0.008	0.132	0.103	0.598
	308	Model 5a (sensitivity): Model 4 + MedDietScore	−0.003 ± 0.006	−0.014; 0.008	0.131	0.099	0.650
	308	Model 5b (sensitivity): Model 4 + total energy intake	−0.007 ± 0.006	−0.019; 0.005	0.140	0.108	0.254
	308	Model 5c (sensitivity): Model 4 + MedDietScore + total energy intake	−0.007 ± 0.006	−0.019; 0.006	0.137	0.102	0.293
	310	Model 6 (sensitivity): Model 4 + education and financial status	−0.003 ± 0.006	−0.014; 0.008	0.131	0.095	0.614

Results are presented as unstandardized b-coefficient, standard error (SE), 95% confidence interval (CI), R^2^, adjusted R^2^ and *p*-value. Antidiabetic treatment was dummy-coded with oral treatment as reference. DPI: Dietary Phytochemical Index; BMI: body mass index. Statistical significance was set at *p*-value < 0.05, marked with bold. No evidence of problematic multicollinearity was observed across the multivariable models (maximum VIF = 1.672 and minimum tolerance = 0.598).

**Table 3:** Multivariable linear regression analysis of fasting plasma glucose according to Dietary Phytochemical Index quartiles.

DPI Quartile	b ± SE	95% CI	*p*-Value
Q1	Reference	-	-
Q2	−6.12 ± 9.51	−24.9; 12.65	0.521
Q3	−15.23 ± 9.17	−33.34; 2.87	0.099
Q4	−20.9 ± 9.11	−38.87; −2.93	**0.023**

Results are presented as unstandardized b-coefficient, standard error (SE), 95% confidence interval (CI) and *p*-value. Analytical sample = 199. Adjusted for age, sex, BMI, physical activity, smoking/vaping status, diabetes duration, antidiabetic treatment and health conditions. Statistical significance was set at *p*-value < 0.05, marked with bold. Q1: lowest quartile <41.3%, Q2: 41.3–50%, Q3: 50–58.4%, Q4: highest quartile >58.4%.

**Table 4:** Exploratory separate multivariable linear regression models assessing the associations between individual DPI food-group components and fasting plasma glucose among 199 adults with type 2 diabetes included in the complete-case analysis.

	DPI Food Group Component	b ± SE per 1% Higher Energy Contribution	95% CI	BH-FDR q	*p*-Value
1	Fruits (% energy)	−1.830 ± 0.999	−3.801; 0.141	0.203	0.069
2	Vegetables (% energy)	−0.440 ± 1.338	−3.080; 2.200	0.872	0.742
3	Legumes (% energy)	0.207 ± 1.286	−2.332; 2.745	0.872	0.872
4	Whole grains (% energy)	−1.875 ± 1.028	−3.904; 0.154	0.203	0.070
5	Nuts/seeds (% energy)	−0.411 ± 0.857	−2.103; 1.281	0.872	0.632
6	Olives and olive oil (% energy)	−0.570 ± 0.346	−1.253; 0.113	0.203	0.101

Results are presented as unstandardized b-coefficient, standard error (SE), 95% confidence interval (CI), Benjamini–Hochberg false discovery rate (BH-FDR) correction and *p*-value. DPI: Dietary Phytochemical Index. All models were adjusted for age, sex, BMI, physical activity, smoking/vaping status, diabetes duration, antidiabetic treatment (as dummy variable with oral treatment as reference) and health conditions.

## Data Availability

Data are unavailable due to privacy or ethical restriction.

## Supplementary Materials

The following supporting information can be downloaded at https://www.mdpi.com/article/10.3390/nu18193130/s1. Table S1. Basic socio-demographic, lifestyle, anthropometric, clinical and dietary characteristics of participants included in the primary complete-case analysis (n = 199) and participants not included because of incomplete data (n = 141). Table S2. Macronutrient composition and absolute energy contribution of DPI food groups across DPI quartiles. Table S3. Multiple linear regression models performed to assess the association between the Dietary Phytochemical Index (DPI) (continuous variable) with fasting plasma glucose levels after excluding n = 29 participants with extreme reported energy intakes (<500 or >3500 kcal/day for women and <800 or >4200 kcal/day for men). Table S4. Sensitivity analysis for the association between DPI and fasting plasma glucose using multiple imputation. Table S5. Multivariable linear regression analysis as sensitivity analysis of fasting plasma glucose according to DPI quartiles using multiple imputation.

## Abbreviations

The following abbreviations are used in this manuscript:

BMI	Body Mass Index
DPI	Dietary Phytochemical Index
FFQ	Food Frequency Questionnaire
HbA1c	Glycosylated Hemoglobin
T2D	Type 2 Diabetes
WHR	Waist-to-Hip Ratio
