# Supplementary material for: Dietary Phytochemical Index and Fasting Plasma Glucose in Adults with Type 2 Diabetes: A Cross-Sectional Study
Source: Nutrients. 2026 Sep 23;18(19):3130. doi: 10.3390/nu18193130 (PMC13666796; doi:10.3390/nu18193130)
Supplement: Supplementary file 1 [file nutrients-18-03130-s001.zip › nutrients-4511051-supplementary.pdf]

Supplementary Table S1. Basic socio-demographic, lifestyle, anthropometric, clinical and dietary characteristics of participants included in the primary complete-case analysis (n = 199) and participants not included because of incomplete data (n = 141).

**Characteristics**	**Included in Model 4 (n=199)**	**Not included in Model 4 (n=141)**	**p**
Age (years)	65.0 ± 11.7	67.1 ± 11.0	0.100
Males, n (%)	107 (54.0)	64 (45.7)	0.130
Education (years)	11.1 ± 4.1	10.2 ± 4.5	0.058
Financial status % (Low / Moderate / Adequate / High)	37.2 / 50.5 / 12.2 / 0	32.5 / 46.4 / 19.2 / 2.0	0.065
Smoking/vaping, yes n (%)	55 (27.5)	43 (30.5)	0.525
IPAQ (MET-min/week)	733 ± 1111	756 ± 865	0.830
BMI (kg/m2)	30.5 ± 6.4	31.0 ± 6.4	0.949
Waist-to-hip ratio	0.97 ± 0.11	0.99 ± 0.10	0.433
Diabetes duration (years)	12.5 ± 9.2	11.8 ± 9.4	0.472
Antidiabetic treatment % (oral antidiabetic treatment / insulin / combination therapy)	61.9 / 7.9 / 30.2	70.7 / 6.7 / 22.7	0.234
Fasting plasma glucose (mg/dL)	131.7 ± 45.5	141.6 ± 39.1	0.387
HbA1c (%)	6.91 ± 1.31	6.77 ± 1.40	0.363
Health conditions (number)	2.42 ± 1.67	2.78 ± 1.76	0.059
MedDietScore (0 – 55)	31.1 ± 4.3	30.3 ± 4.5	0.099
DPI (%)	49.2 ± 12.5	50.9 ± 11.4	0.192
Carbohydrate intake (g/d)	198 ± 89	195 ± 77	0.728
Protein intake (g/d)	122 ± 57	115 ± 43	0.216
Fat intake (g/d)	157 ± 47	157 ± 42	0.944
Total Energy Intake (kcal/d)	2634 ± 881	2603 ± 724	0.731

Data are presented as mean ± standard deviation for continuous variables and frequencies for categorical variables. Between-group comparisons were performed using independent-sample t-test for continuous variables and the chi-square test for categorical values. Statistical significance was set at p-value < 0.05, marked with bold. DPI: Dietary Phytochemical Index; IPAQ: International Physical Activity Questionnaire; BMI: Body Mass Index; HbA1c: Glycated Haemoglobin.

Supplementary Table S2. Macronutrient composition and absolute energy contribution of DPI food groups across DPI quartiles

	**Total (N = 340)**	**Q1** (n = 85)	**Q2** (n = 85)	**Q3** (n = 85)	**Q4** (n = 85)	**p**
Carbohydrates (% energy)	29.6 ± 6.5	32.4 ± 7.6	30.3 ± 5.9	28.3 ± 6.0	27.3 ± 5.2	**<0.001**
Protein (% energy)	17.9 ± 4.2	19.4 ± 5.2	18.3 ± 4.4	17.3 ± 3.6	16.6 ± 3.0	**<0.001**
Fat (% energy)	54.4 ± 7.0	49.5 ± 7.0	53.0 ± 5.5	56.2 ± 6.2	59.0 ± 5.5	**<0.001**
Total DPI numerator (kcal/d)	1268 ± 352	1084 ± 400	1280 ± 335	1317 ± 311	1393 ± 281	**<0.001**
Fruits (kcal/d)	139 ± 93	127 ± 85	133 ± 80	146 ± 100	149 ± 106	0.379
Vegetables (kcal/d)	141 ± 79	143 ± 83	155 ± 89	133 ± 72	135 ± 68	0.259
Legumes (kcal/d)	97 ± 70	98 ± 77	99 ± 75	90 ± 63	100 ± 67	0.774
Whole grains (kcal/d)	96 ± 94	74 ± 92	90 ± 99	104 ± 89	117 ± 90	**0.016**
Nuts/seeds (kcal/d)	75 ± 119	49 ± 80	71 ± 99	92 ± 139	88 ± 142	0.070
Olives/olive oil (kcal/d)	720 ± 213	593 ± 296	733 ± 185	753 ± 158	803 ± 107	**<0.001**

Values are mean ± SD. DPI numerator equals the sum of daily energy from fruits, vegetables, legumes, whole grains, nuts/seeds, and olives/olive oil. DPI: Dietary Phytochemical Index. Macronutrient percentages were calculated using 4 kcal/g for carbohydrate and protein and 9 kcal/g for fat. P-values derived from one-way ANOVA. Statistical significance was set at p-value < 0.05, marked with bold.

Supplementary Table S3. Multiple linear regression models performed to assess the association between the Dietary Phytochemical Index (DPI) (continuous variable) with fasting plasma glucose levels after excluding n = 29 participants with extreme reported energy intakes (<500 or >3500 kcal/day for women and <800 or >4200 kcal/day for men).

Models	b ± SE per 1 % increase in DPI	95% CI	R^2	Adjusted R^2	p
**Model 1** (crude): DPI	-0.869 ± 0.275	-1.411; -0.327	0.052	0.047	**0.002**
**Model 2:** Model 1 + age, sex	-0.869 ± 0.281	-1.423; -0.315	0.054	0.039	**0.002**
**Model 3**: Model 2 + BMI, physical activity, smoking/vaping status	-0.836 ± 0.285	-1.399; -0.272	0.071	0.039	**0.004**
**Model 4:** Model 3 + diabetes duration, antidiabetic treatment, health conditions	-0.827 ± 0.301	-1.422; -0.233	0.116	0.061	**0.007**
**Model 5a (sensitivity):** Model 4 + MedDietScore	-0.771 ± 0.315	-1.393; -0.149	0.118	0.057	**0.015**
**Model 5b (sensitivity):** Model 4 + Total energy intake	-0.787 ± 0.318	-1.414; -0.159	0.117	0.056	**0.014**
**Model 5c (sensitivity):** Model 4 + MedDietScore + total energy intake	-0.702 ± 0.338	-1.370; -0.034	0.120	0.053	**0.04**
**Model 6 (sensitivity):** Model 4 + education and financial status	-0.884 ± 0.290	-1.457; -0.312	0.125	0.058	**0.003**

Results are presented as unstandardized b-coefficient, standard error (SE), 95% Confidence Interval (CI), R^2, adjusted R^2 and p-value. Antidiabetic treatment was dummy-coded with oral treatment as reference. DPI: Dietary Phytochemical Index; BMI: Body mass index. Statistical significance was set at p-value < 0.05, marked with bold.

Supplementary Table S4. Sensitivity analysis for the association between DPI and fasting plasma glucose using multiple imputation.

Models	Multiple-imputation analysis		
	**n***	**Pooled b (95% CI)**	**p**
**Model 1** (crude): DPI	340	-0.572 (-1.010; -0.134)	**0.011**
**Model 2:** Model 1 + age, sex	340	-0.554 (-0.997; -0.110)	**0.015**
**Model 3**: Model 2 + BMI, physical activity, smoking/vaping status	340	-0.559 (-1.003; -0.114)	**0.014**
**Model 4:** Model 3 + diabetes duration, antidiabetic treatment, health conditions	340	-0.518 (-0.958; -0.079)	**0.021**
**Model 5a (sensitivity):** Model 4 + MedDietScore	340	-0.524 (-0.977; -0.071)	**0.024**
**Model 5b (sensitivity):** Model 4 + Total energy intake	340	-0.582 (-1.101; -0.063)	**0.028**
**Model 5c (sensitivity):** Model 4 + MedDietScore + total energy intake	340	-0.594 (-1.143; -0.046)	**0.034**
**Model 6 (sensitivity):** Model 4 + education and financial status	340	-0.526 (-0.962; -0.089)	**0.019**

Results are presented as unstandardized b-coefficient, standard error (SE), 95% Confidence Interval (CI), and p-value. *The multiple-imputation analyses used 50 imputed datasets and included all 340 participants in each model; fasting plasma glucose was observed for 216 participants and imputed for 124. Estimates and standard errors were pooled using Rubin's rules. Antidiabetic treatment was dummy-coded with oral treatment as reference. DPI: Dietary Phytochemical Index; BMI: Body mass index.

Supplementary Table S5. Multivariable linear regression analysis as sensitivity analysis of fasting plasma glucose according to DPI quartiles using multiple imputation.

DPI quartile	**b ± SE**	**95% CI**	**p-value**
Q1	Reference	-	-
Q2	-4.41 ± 7.34	-18.9; 10.1	0.549
Q3	-12.8 ± 7.29	-27.2; 1.53	0.080
Q4	-15.9 ± 7.56	-30.8; -0.94	**0.037**
Linear trend across quartiles[1]	-0.60 ± 0.26	-1.11; -0.09	**0.021**
Linear trend across quartiles after exclusion of implausible energy intakes[2]	-0.59 ± 0.25	-1.07; -0.11	**0.017**

Results are presented as unstandardized b-coefficient, standard error (SE), 95% Confidence Interval (CI) and p-value. Adjusted for age, sex, BMI, physical activity, smoking/vaping status, diabetes duration, antidiabetic treatment and health conditions. The multiple-imputation analyses used 50 imputed datasets; fasting plasma glucose was observed for 216 participants and imputed for 124. [1]The median DPI value within each quartile was entered as a continuous variable. [2]Implausible energy intake was defined as <500 or >3500 kcal/day for women and <800 or >4200 kcal/day for men; 29 participants were excluded. Quartile cut-offs were Q1 <41.3%, Q2 41.3–50.0%, Q3 >50.0–58.4% and Q4 >58.4%. Statistical significance was set at p-value < 0.05, marked with bold. Q1: Lowest quartile <41.3%, Q2: 41.3 – 50%, Q3: 50 – 58.4%, Q4: Highest quartile >58.4%.
